# Psychometric validation of the EuroQoL 5-Dimension 5-Level (EQ-5D-5L) in Chinese patients with adolescent idiopathic scoliosis

**DOI:** 10.1186/s13013-016-0083-x

**Published:** 2016-08-04

**Authors:** Prudence Wing Hang Cheung, Carlos King Ho Wong, Dino Samartzis, Keith Dip Kei Luk, Cindy Lo Kuen Lam, Kenneth Man Chee Cheung, Jason Pui Yin Cheung

**Affiliations:** 1 Department of Orthopaedics and Traumatology, The University of Hong Kong, Queen Mary Hospital, 5/F, Professorial Block, Pokfulam, Hong Kong, SAR China; 2 Department of Family Medicine and Primary Care, The University of Hong Kong, Hong Kong, SAR China

**Keywords:** Quality of life, Psychometrics, EQ-5D-5L, Validity, Reliability, Adolescent idiopathic scoliosis, Chinese

## Abstract

**Background:**

Scoliosis is a common spinal deformity that occurs often during adolescence. Previous studies suggested that adolescent idiopathic scoliosis (AIS) patients can have various aspects of their lives being affected, due to disease presentation and/or treatment received. It is important to define a reliable instrument based on which the affected patients’ health-related quality of life can be assessed. This study aims to assess the validity, reliability and sensitivity of the EuroQoL 5-dimension 5-level (EQ-5D-5L) in Chinese patients with AIS.

**Methods:**

Adolescent idiopathic scoliosis patients of Chinese descent were prospectively recruited to complete both the traditional Chinese versions of the EQ-5D-5L and the refined Scoliosis Research Society-22 (SRS-22r) questionnaires. Patients’ demographic profiles and corresponding clinical parameters including treatment modalities, spinal curve pattern and magnitude, and duration of bracing were recorded. Telephone interviews were then conducted at least two weeks later for the assessment of test-retest reliability. Statistical analysis was performed: construct validity of the EQ-5D-5L domains were assessed using Spearman’s correlation test against the SRS-22r; whereas intra-class correlation coefficient (ICC) was used to assess the test-retest reliability, and agreement over the test-retest period was expressed in percentages. Also, the sensitivity of the EQ-5D-5L in differentiating various clinical known groups was determined by effect size, independent t-test and analysis of variance.

**Results:**

A total of 227 AIS patients were recruited. Scores of domains of the EQ-5D-5L correlated significantly (r: 0.57-0.74) with the scores of the SRS-22r domains that were intended to measure similar constructs, supporting construct validity. The EQ-5D-5L domain responses and utility scores showed good test-retest reliability (ICC: 0.777; agreement: 76.4 -98.1 %). Internal consistency was good (Cronbach’s α: 0.78) for the EQ-5D-5L utility score. The EQ-5D-5L utility score was sensitive in detecting differences between subjects who had different treatment modalities and bracing duration, but not for curve pattern and its magnitude.

**Conclusions:**

The EQ-5D-5L is found to be a valid, reliable and sensitive measure to assess the health-related quality of life in Chinese AIS patients. This potentiates the possibility of utilizing the EQ-5D-5L to estimate AIS patients’ health-related quality of life, based on which the outcome of various treatment options can eventually be evaluated.

**Electronic supplementary material:**

The online version of this article (doi:10.1186/s13013-016-0083-x) contains supplementary material, which is available to authorized users.

## Background

Scoliosis can be defined as a torsional spinal deformity, in which the 3-dimensional geometry of the spine is changed as a result of the combination of a translation and rotation of variable number of vertebrae [1]. A majority of scoliosis is idiopathic and presents during adolescence [2]. These patients with adolescent idiopathic scoliosis (AIS) often present at variable curve magnitudes upon the first consultation and the curvature may progress depending on the initial magnitude of curve and status of skeletal maturity [3, 4]. The natural history may also be affected by the introduction of any intervention such as bracing before patients have reached skeletal maturity [5].

Besides the obvious radiographic differences in curve magnitude, any treatment option can only truly demonstrate benefit with superior patient-perceived outcome measures. It is thus necessary to explore patients’ quality of life. This is particularly important in AIS as previous reports suggest that these patients experience relatively poorer psychosocial functioning, self-perception of body image, and health-related quality of life versus their non-scoliotic peers [6]. When compared to their healthy peers, AIS patients undergoing brace treatment may be negatively affected in terms of psychosocial well-being [7, 8]. Among various treatment modalities, AIS patients with observation may experience a better score for body image and quality of life than braced patients [9, 10]. On the contrary, there are studies suggesting that there are no differences in the quality of life between patients treated with bracing and those under monitoring only [11]; and even between braced/operated patients and the general population in the long-term [12].

The reported evidence here suggest that AIS can affect the health-related quality of life (HRQOL) of the affected adolescents, which can be variable depending on the severity of disease presentation, and different treatment options. With the appropriate indications for treatment in place, healthcare providers may be able to improve the HRQOL of AIS patients with timely interventions. For instance, patients may benefit from interventions, such as psychological therapy accompanying the administration of bracing. This can improve the self-perception of body image, which is a barrier to the initiation and continuation of brace treatment [13], and ultimately enhances brace compliance. Therefore, a reliable instrument tailored for AIS is desirable to assess the physical ability, psychological well-being and psychosocial functioning of these patients. Moreover, the instrument serves as an indicator of how these factors can impact the HRQOL of the AIS population in general.

In fact, validated outcome measurements, together with systemic reviews based on clinical trials, form the scientific framework of evidence-based medicine (EBM), which is used to guide clinical practice [14]. Evidence-based medicine can be defined as an integration of the best research evidence with clinical expertise and patient values [15]. Ultimately, the goal of EBM is to provide scientific information to clinicians to improve the quality of healthcare by taking into account cost, ethics and safety. Adoption of EBM to clinical practice depends on the quality of evidence (i.e. from the validated outcome measurements and systemic reviews on clinical trials), and the willingness of the clinician to apply that evidence to their practice [14]. Therefore, it is of utmost importance to utilize an effective and appropriate objective outcome measure for the assessment of patient values and their quality of life. This can be accomplished by the use of structured questionnaires to measure an individual's perception of his/her physical, mental and social ability to function [16].

Several systematic reviews [6, 17, 18] have summarized that various instruments can assess the HRQOL of AIS patients, and are primarily classified into two main categories: generic and condition-specific instruments. As generic instruments capture a very broad range of health statuses, condition-specific measures specifically assess the special states and functions of a particular disease in greater details than generic measures [16], with more responsiveness in detecting important changes over time, and better sensitivity in discovering subtle effects of interventions [10, 19]. However, disease-specific instruments can only focus on known and anticipated consequences [20, 21]. These instruments do not allow obvious comparisons across populations of different diseases, and between outcomes of different treatments for patients with various health problems [16]. On the contrary, generic measures give health state utility values that permit comparisons between patient groups [22], or cost-effectiveness comparisons between different treatment modalities for various diseases [23]. It can be used to generate ‘normative values’ with which patients with health problems can be compared [16]. Despite generic measures may have value in detecting unexpected positive or negative effects of an intervention [24], its non-specific nature can have reduced sensitivity in detecting changes caused by interventions in relevance to any one illness, especially in clinical trials. Generic measure allow broad applicability across specialties or populations but is multi-domain. This poses a risk of results misinterpretation if improvement in only a single domain is reported as general improvement in quality of life and may distort general scoring [25].

The refined Scoliosis Research Society-22 (SRS-22r) was originally developed for aiming at measuring spine-specific HRQOL of adolescent or adult patients with scoliosis. Given that two domains (self-image and satisfaction with management) of the SRS-22r are relevant and only specific to scoliosis patients, the measured constructs in the SRS-22r instrument may not fully overlap with generic instruments. Previous studies [26, 27] administering both the generic and spine-specific instruments suggest that self-image and satisfaction with management are poorly correlated with domains of generic instruments. This is the case with commonly used instruments like the EuroQoL 5-dimension (EQ-5D) [26] and the 36-Item Short Form Health Survey (SF-36) [27], whose domains do not relate well to spine-specific instruments. Furthermore, generic instruments allow head-to-head comparisons among different health conditions, particularly for the EQ-5D, as a preference-based measure which enables calculation of quality-adjusted life years (QALYs) in economic evaluation. As such, the spine-specific HRQOL instruments may not supersede the generic instruments among AIS patients. Therefore, the aim of the present study was to assess the validity, reliability and sensitivity of the EQ-5D in Chinese AIS patients.

## Methods

### Subjects and setting

Convenience sampling of patients with histological proof of AIS patients of Chinese ethnicity were recruited between August and October 2015 at the Duchess of Kent Children’s Hospital in Hong Kong. Exclusion criteria included patients with non-idiopathic scoliosis (congenital/neuromuscular), who could not understand traditional Chinese, refused to participate or were physically or mentally unfit. This study was ethically approved by the local institutional review board.

Subjects who consented were asked to answer a structured questionnaire which consisted of the EQ-5D-5L questionnaire (Hong Kong (traditional Chinese) EQ-5D-5L version) and the traditional Chinese version of the SRS-22r questionnaire. Half of the subjects were asked to fill in and complete the SRS-22r questionnaire first prior to being given the EQ-5D-5L, and the other half were given the questionnaires in the reversed order.

Demographic data of patients and clinical data at the time of visit were collected. A spine surgeon performed the consultation and radiographic measurement as usual, without prior knowledge of the conduction of questionnaires. The Cobb angle [28] was measured on the whole spine radiograph taken at that appointment and were recorded. Also, the curvatures were classified using the modified Lenke classification system [29] which included six curve types: type 1 (main thoracic), type 2 (double thoracic), type 3 (double major; thoracic curve larger than lumbar curve), type 4 (triple major), type 5 (thoracolumbar or lumbar curve), type 6 (double major; thoracolumbar or lumbar curve larger than thoracic curve). Treatment modalities of whether patients were undergoing observation, bracing, bracing followed by surgery and those who had corrective surgery but presented for regular review, were retrieved from subjects' medical records.

All subjects were scheduled for a telephone interview conducted by a single research personnel in a random order, at least two weeks after their baseline interview. This follow-up interview consisted of administering the two questionnaires in the same order as at baseline. This was structured to assess the test-retest reliability of our study instruments.

### Study instruments

#### EuroQoL 5-dimension 5-level (EQ-5D-5L) (Additional file 1)

The EQ-5D-5L is a generic health status measure developed by the EuroQol Group for measurement of quality of daily life [30], providing descriptions of five dimensions of health status. It is an instrument enabling a quantitative expression of the individual’s values and preferences regarding overall health status [16, 31]. Being a utility measure, the EQ-5D-5L plays an important role in both clinical and economic appraisal, for instance in the assessment of social value of different healthcare interventions by means of cost-utility analysis[32], and its possible use as decision-aids in individual patient care where patients having difficulties deciding between treatment options [33].

The EQ-5D-5L has five domain scales (mobility, self-care, usual activities, pain and discomfort, and anxiety and depression) and five levels for each domain. Since the Chinese-specific EQ-5D-5L value set / tariff is currently not available, we applied a two-step indirect approach to estimate the EQ-5D-5L scores applicable for Chinese population, as adopted in previous studies [34]. The first step was the application of an indirect interim mapping method [35]. The EQ-5D-5L health status was transformed to the EQ-5D-3L health status according to the transition probability matrix. Finally, the EQ-5D-3L health status were scored according to a recently developed Chinese-specific the EQ-5D-3L value set ranging from −0.149 for the worst health status (‘33333’) to 1 for the full health (‘11111’) [36]. Since the EQ-5D-5L has 5 items, each digit in the five digit codes refers to the status of each dimension, ranging from 1 for no problem, to 5 for severe problem. For example, the five digit of ‘11111’ implies to a health status with no problems in the 5 dimensions, scoring 1 being the best score with no problem in each domain listed in the order of: mobility = 1, self-care =1, usual activities =1, pain and discomfort =1, anxiety and depression =1. A higher score in the EQ-5D-5L indicated better HRQOL.

### Refined Scoliosis Research Society-22 (SRS-22r) (Additional file 2)

The SRS-22r is a simple and valid spine-specific health-related quality of life instrument developed by the Scoliosis Research Society. It provides an insight into the idiopathic scoliosis patient’s perception of his/her condition [37]. The SRS-22r is a refinement of the previous SRS-22 questionnaire, with a minor revision (i.e. Question 18- related to going out, and a concern over Question 15 – related to financial considerations), it makes gathering of longitudinal HRQOL information from adolescence through adulthood possible [38].

The SRS-22r had 22 items grouped into five subscales. The domains covered were: Function (5 items), Pain (5 items), Self-image/appearance (5 items), Mental Health (5 items) and Satisfaction with Management (currently undergoing or had been performed – 2 items). The sum of domain scores gave the overall SRS-22r total score with a range from 0 to 5. Patients were asked to indicate the stage of undergoing treatment, whether they were present for initial consultation, regular follow-up without intervention, bracing, immediately pre-operative, or postoperative. The SRS-22r questionnaire had been previously validated in the Hong Kong Chinese scoliosis population [39].

### Statistical analysis

Descriptive statistics including mean, standard deviation (±SD) and percentage of floor and ceiling of domain and total scores were calculated. At least 15 % of patients achieving the lowest or highest possible score was considered as presence of floor or ceiling effect, respectively [40]. The construct validity of the EQ-5D-5L domain was assessed using Spearman’s correlation test against the SRS-22r domain scores holding similar constructs.

The internal consistency was assessed by Cronbach’s alpha using a value >0.7 to indicate adequate internal consistency [41]. Test-retest reliability was assessed by examining the weighted kappa for five individual domain responses and the intra-class correlation coefficient (ICC) for the EQ-5D-5L score over the 2-week period. An ICC of ≥0.7 was used to indicate good reproducibility of the EQ-5D-5L score [40]. A weighted Kappa of <0.2 was interpreted as poor agreement of individual domain responses between two assessments, 0.21-0.4 as fair, 0.41-0.6 as moderate, 0.61-0.8 as good and ≥0.8 as very good [42].

The sensitivity of the EQ-5D-5L score was determined by performing known group comparisons by effect size, independent t-test and analysis of variance, where appropriate. Cohen's effect size was calculated as the difference between mean scores, divided by pooled SD. Comparisons of known clinical groups were (i) Observation treatment versus bracing or surgery; (ii) Observation treatment versus bracing only; (iii) Bracing versus surgery; (iv) Duration of bracing: for less than, or more than one year; (v) Curve Pattern: Modified Lenke Classification type 1/2 (thoracic curves only) versus type 5 (lumbar curves only) versus type 3/4/6 (thoracic and lumbar curves); (vi) Curve magnitude: Cobb angle ≤40° versus >40°.

Data analyses were conducted using SPSS Windows 23.0 (IBM SPSS Inc., Chicago, IL, USA) and STATA version 13.0 (StataCorp LP. College Station, Texas, U.S.). *P*-value <0.05 was statistically significant.

## Results

A total of 227 patients with AIS were recruited to participate in completing both the SRS-22r and the EQ-5D-5L questionnaires. All the patients gave consent and agreed on participation. Hence a total of 227 eligible patients were included in the psychometric validation of the EQ-5D-5L. The mean age was 15.6 (±SD: 4.5) years, 74.9 % of female, and 9.7 % of severe curvature with Cobb angle of >40°. About 62 % were under Observation management with regular follow-up while the remaining subjects were braced before (5.7 %), undergoing bracing (0.8 %) and underwent surgery before (9.3 %). Baseline characteristics of AIS patients are shown in Table 1.

**Table 1:** Baseline characteristics of adolescent idiopathic scoliosis patients

	Total (*N* = 227)	
Characteristics	*n*	%
Age (years, Mean ± SD)	15.6 ± 4.5	
Gender		
Male	57	25.1 %
Female	170	74.9 %
Cobb Angle	25.0 ± 11.4	
≤40°, Mild or moderate	205	90.3 %
>40°, Severe	22	9.7 %
Treatment modality		
Observation with regular follow-up	139	61.2 %
Braced before	13	5.7 %
Bracing	54	23.8 %
Surgery	21	9.3 %
Duration of bracing		
<1 year	20	37.0 %
≥1 year	34	63.0 %
Modified Lenke Classification		
Thoracic curve (Types 1/2)	86	37.9 %
Lumbar curve (Type 5)	38	16.7 %
Thoracic & Lumbar curve (Types 3/4/6)	103	45.4 %

*N/n* number of subjects, *SD* standard deviation

Table 2 summarizes the mean, standard deviations, floor and ceiling effects of the EQ-5D-5L and SRS-22r subscale scores, and distribution of the EQ-5D-5L domain responses. No significant floor effect were observed for all HRQOL scores but the EQ-5D-5L (66 %), Function/Activity (67 %), Pain (45 %) and Mental Health (32 %) subscale scores reflected significant ceiling effect. Over 70 % of patients perceived as “no problems” in all EQ-5D-5L domains (70.0 - 96.3 %). No patients responded with “extreme problems” in Mobility, Self-care, Usual Activities, and Depression/Anxiety; and “unable to” in all EQ-5D-5L dimensions.

**Table 2:** Descriptive statistics of domain response and scores of the EuroQoL 5-dimension 5-level (EQ-5D-5L) and the Refined Scoliosis Research Society-22 (SRS-22r)

Domain/Total score	Mean	Standard Deviation	Observed Range	Theoretical Range	Floor (%)	Ceiling (%)
EQ-5D-5L	0.93	0.11	0.34-1.00	−0.15-1.00	0	66
SRS-22r						
Function/activity	4.8	0.42	2.6-5.0	0.0-5.0	0	67
Pain	4.7	0.44	1.8-5.0	0.0-5.0	0	45
Appearance	3.9	0.64	2.0-5.0	0.0-5.0	0	9
Mental Health	4.4	0.58	2.6-5.0	0.0-5.0	0	32
Satisfaction with management	1.1	1.85	0.0-5.0	0.0-5.0	0	5
Total	4.4	0.42	2.6-5.0	0.0-5.0	0	2
EQ-5D-5L Dimension (%)	No problems	Slight problems	Moderate problems	Severe problems	Unable to	
Mobility	93.4 %	5.3 %	1.3 %	0.0 %	0.0 %	
Self-care	96.0 %	3.1 %	0.9 %	0.0 %	0.0 %	
Usual activities	85.9 %	12.8 %	1.3 %	0.0 %	0.0 %	
Pain/discomfort	70.0 %	28.2 %	0.9 %	0.9 %	0.0 %	
Depression/anxiety	79.3 %	17.6 %	3.1 %	0.0 %	0.0 %	

Test-retest reliability of the EQ-5D-5L and SRS-22r are shown in Table 3. There were 20 patients who failed to comply with telephone interviews, and one patient was eliminated at test-retest due to the change in treatment modality from preoperative regular follow-up to postoperative hospitalization. Among 106 (83.5 %) patients assessed in both baseline and 2-week retest interviews, the mean interval between interviews was 19.7 days (range: 15–36 days). The ICC of the EQ-5D-5L and SRS-22r subscales and overall scores exceeded 0.7. Agreement of the EQ-5D-5L domain responses between two interviews ranged from 76.4 % in Pain/discomfort to 98.1 % in Self-care. Cronbach’s alpha coefficient was 0.78 in the EQ-5D-5L score, indicating acceptable internal consistency reliability.

**Table 3:** Test-retest reliability of domain and total scores of the EuroQoL 5-dimension 5-level (EQ-5D-5L) and the Refined Scoliosis Research Society-22 (SRS-22r)

	Intra-class correlation				
Subscale/Total score	n	Estimate	95 % CI		
EQ-5D-5L score	106	0.78	0.67 -	0.85	
SRS-22r					
Function/activity	105	0.89	0.84 -	0.92	
Pain	105	0.85	0.78 -	0.90	
Appearance	105	0.88	0.82 -	0.92	
Mental Health	105	0.84	0.77 -	0.89	
Satisfaction with management	105	0.94	0.91 -	0.96	
Total	105	0.93	0.90 -	0.95	
EQ-5D-5L Domain	*n*	Weighted Kappa	95 % CI		Agreement
Mobility	106	.19	.08 -	.29	92.5 %
Self-care	106	.66	.48 -	.84	98.1 %
Usual activities	106	.52	.35 -	.69	88.7 %
Pain/discomfort	106	.45	.28 -	.63	76.4 %
Depression/anxiety	106	.67	.49 -	.84	90.6 %

*n* number of subjects, *CI* confidence interval

Correlations between the EQ-5D-5L domain responses and SRS-22r domain scores are depicted in Table 4. Those patients perceived as “no problems” in the EQ-5D-5L domain had significantly higher Function/Activity, Pain, Appearance and total scores of the SRS-22r than those perceived as having “any problems”. However, those patients having “no problems” in Self-care, Usual Activities and Pain/discomfort had significantly lower satisfaction with management than those having “any problems”. Furthermore, the EQ-5D-5L score had a strong correlation with Function/Activity (*r* = 0.715) and total scores (*r* = 0.735) of the SRS-22r, and a moderate correlation with Pain (*r* = 0.594) and Appearance (*r* = 0.512) scores with *p* < 0.001.

**Table 4:** Correlation between the EuroQoL 5-dimension 5-level (EQ-5D-5L) dimension and the Refined Scoliosis Research Society-22 (SRS-22r) domain scores

	SRS-22r Domain and total scores											
EQ-5D-5L Domain	Function/activity		Pain		Appearance		Mental Health		Satisfaction with management		Total	
	Mean	*P*-value	Mean	*P*-value	Mean	*P*-value	Mean	*P*-value	Mean	*P*-value	Mean	*P*-value
Mobility		<0.001		<0.001		<0.001		<0.001		0.168		<0.001
No problems	4.84		4.72		3.99		4.47		1.03		4.50	
Any problems	3.86		3.90		3.10		3.63		1.71		3.59	
Self-care		<0.001		<0.001		<0.001		<0.001		0.032		<0.001
No problems	4.81		4.69		3.96		4.45		1.02		4.47	
Any problems	4.00		4.09		3.24		3.69		2.33		3.74	
Usual activities		<0.001		<0.001		<0.001		<0.001		<0.001		<0.001
No problems	4.91		4.76		4.06		4.54		0.88		4.56	
Any problems	3.98		4.09		3.19		3.71		2.22		3.73	
Pain/discomfort		<0.001		<0.001		<0.001		<0.001		0.006		<0.001
No pain/discomfort	4.91		4.81		4.07		4.56		0.85		4.58	
Any pain/discomfort	4.47		4.32		3.62		4.10		1.58		4.12	
Depression/anxiety		<0.001		<0.001		<0.001		<0.001		0.796		<0.001
No problems	4.87		4.76		4.09		4.58		1.05		4.56	
Any problems	4.42		4.31		3.34		3.80		1.13		3.96	
	*r*	*P*-value	*r*	*P*-value	*r*	*P*-value	*r*	*P*-value	*r*	*P*-value	*r*	*P*-value
EQ-5D-5L Score	0.72	<0.001	0.59	<0.001	0.51	<0.001	0.57	<0.001	−0.14	0.034	0.74	<0.001

Sensitivity of the EQ-5D-5L in differentiating known clinical groups are displayed in Table 5. The EQ-5D-5L and SRS-22r scores were able to detect statistical differences in treatment modalities (i.e. observation management versus bracing or surgery, or observation management versus bracing). Statistical differences in the EQ-5D-5L was detected between bracing patients with duration of less <1 year and ≥1 year but the SRS-22r did not. Cobb angle and curve type in terms of the modified Lenke classification were not associated with the EQ-5D-5L and SRS-22r scores, with the exception that the patients with severe curvature had worse mental health than those with mild or moderate curvature. No differences in the EQ-5D-5L and SRS-22r scores, apart from Appearance and Satisfaction with Management, between patients undergoing bracing and surgery were observed.

**Table 5:** Sensitivity of differentiating known clinical groups

	Treatment (observation versus bracing or surgery)							Treatment (observation versus bracing)						
	Observation (*n* = 139)		Bracing/Surgery (*n* = 88)					Observation (*n* = 139)		Bracing (*n* = 67)				
	Mean	SD	Mean	SD	P-value*	ES^a^		Mean	SD	Mean	SD	P-value*	ES^a^	
EQ-5D-5L	0.96	0.07	0.89	0.15	<0.001	0.66	EQ-5D-5L	0.96	0.07	0.89	0.14	<0.001	0.62	
SRS-22r							SRS-22r							
Function/activity	4.93	0.20	4.53	0.55	<0.001	0.95	Function/activity	4.93	0.20	4.58	0.50	<0.001	0.92	
Pain	4.79	0.29	4.48	0.56	<0.001	0.70	Pain	4.79	0.29	4.52	0.49	<0.001	0.66	
Appearance	4.03	0.55	3.78	0.74	0.004	0.39	Appearance	4.03	0.55	3.68	0.68	<0.001	0.57	
Mental Health	4.49	0.51	4.31	0.67	0.028	0.29	Mental Health	4.49	0.51	4.32	0.63	0.038	0.30	
Satisfaction with management	0.25	1.02	2.37	2.01	<0.001	−1.33	Satisfaction with management	0.25	1.02	2.13	2.01	<0.001	−1.18	
Total	4.56	0.28	4.25	0.52	<0.001	0.73	Total	4.56	0.28	4.25	0.48	<0.001	0.78	
	Treatment (Bracing versus Surgery)							Duration of Bracing						
	Bracing (*n* = 67)		Surgery (*n* = 21)					<1 year (*n* = 34)		≥1 year (*n* = 20)				
	Mean	SD	Mean	SD	P-value*	ES^a^		Mean	SD	Mean	SD	P-value*	ES^a^	
EQ-5D-5L	0.89	0.14	0.86	0.18	0.372	0.21	EQ-5D-5L	0.82	0.15	0.91	0.14	0.023	−0.66	
SRS-22r							SRS-22r							
Function/activity	4.58	0.50	4.38	0.69	0.156	0.33	Function/activity	4.40	0.52	4.58	0.51	0.226	−0.34	
Pain	4.52	0.49	4.33	0.73	0.184	0.30	Pain	4.54	0.34	4.48	0.58	0.657	0.14	
Appearance	3.68	0.68	4.11	0.83	0.021	−0.56	Appearance	3.60	0.74	3.67	0.62	0.732	−0.10	
Mental Health	4.32	0.63	4.30	0.83	0.924	0.02	Mental Health	4.27	0.65	4.28	0.66	0.972	−0.01	
Satisfaction with management	2.13	2.01	3.15	1.84	0.046	−0.53	Satisfaction with management	2.43	2.12	2.52	1.96	0.875	−0.04	
Total	4.25	0.48	4.25	0.67	0.991	0.002	Total	4.19	0.49	4.22	0.50	0.780	−0.08	
	Cobb angle (mild or moderate versus severe)							Modified Lenke Classification						
	≤40° (*n* = 205)		>40° (*n* = 20)					Thoracic curve (*n* = 86)		Lumbar curve (*n* = 38)		Thoracic & Lumbar curve (*n* = 103)		
	Mean	SD	Mean	SD	*P*-value*	ES^a^		Mean	SD	Mean	SD	Mean	SD	*P*-value*
EQ-5D-5L	0.93	0.11	0.91	0.12	0.402	0.19	EQ-5D-5L	0.92	0.13	0.93	0.11	0.95	0.10	0.271
SRS-22r							SRS-22r							
Function/activity	4.78	0.41	4.69	0.52	0.349	0.20	Function/activity	4.73	0.44	4.81	0.39	4.79	0.42	0.522
Pain	4.68	0.43	4.55	0.53	0.216	0.27	Pain	4.64	0.39	4.69	0.38	4.68	0.50	0.753
Appearance	3.96	0.63	3.72	0.76	0.116	0.34	Appearance	3.94	0.70	3.84	0.58	3.97	0.61	0.583
Mental Health	4.45	0.55	4.16	0.81	0.037	0.41	Mental Health	4.41	0.62	4.36	0.61	4.45	0.55	0.703
Satisfaction with management	1.09	1.84	0.90	1.47	0.662	0.11	Satisfaction with management	1.10	1.86	0.99	1.82	1.07	1.77	0.949
Total	4.46	0.40	4.25	0.57	0.032	0.42	Total	4.42	0.45	4.42	0.37	4.46	0.41	0.788

* *P*-value of independent t-test or analysis of variance test, where appropriate

^a^Cohen’s effect size was calculated as the difference between mean scores, divided by pooled SD

*N* number of subjects, *SD* standard deviation, *ES* effect size

Moreover, the profile of the studied population is presented in Table 6. The EQ-5D-5L was able to differentiate patients undergoing various treatment (Observation versus Bracing/Surgery or Observation versus Bracing), based on the domains of Mobility, Self-care, Usual activities and Pain/discomfort (*p* ≤ 0.001). The EQ-5D-5L was also able to differentiate among patients who were undergoing bracing based on the duration of bracing, with the most effective, significant domain being Pain/discomfort with 70 % versus 35.3 % of patients for duration of < 1 year and ≥1 year respectively. However, the EQ-5D-5L cannot differentiate among patients on the basis of the pattern/type (modified Lenke classification) and the magnitude of curvature.

**Table 6:** Correlation between the EuroQoL 5-dimension 5-level (EQ-5D-5L) Dimension and known clinical groups

	Treatment (Observation versus bracing or surgery)			Treatment (Observation versus bracing)			
% in any problems	Observation (*n* = 139)	Bracing/Surgery (*n* = 88)	*P*-value*	Observation (*n* = 139)	Bracing (*n* = 67)	*P*-value*	
Mobility	0.7 %	15.9 %	<0.001	0.7 %	14.9 %	<0.001	
Self-care	0.0 %	10.2 %	<0.001	0.0 %	10.4 %	<0.001	
Usual activities	1.4 %	34.1 %	<0.001	1.4 %	34.3 %	<0.001	
Pain/discomfort	20.1 %	45.5 %	<0.001	20.1 %	41.8 %	0.001	
Depression/anxiety	18.0 %	25.0 %	0.204	18.0 %	26.9 %	0.142	
	Treatment (Bracing versus Surgery)			Duration of Bracing			
% in any problems	Bracing (*n* = 67)	Surgery (*n* = 21)	*P*-value*	<1 year (*n* = 34)	≥1 year (*n* = 20)	*P*-value*	
Mobility	14.9 %	19.0 %	0.652	30.0 %	11.8 %	0.096	
Self-care	10.4 %	9.5 %	0.903	25.0 %	5.9 %	0.043	
Usual activities	34.3 %	33.3 %	0.933	60.0 %	29.4 %	0.027	
Pain/discomfort	41.8 %	57.1 %	0.218	70.0 %	35.3 %	0.014	
Depression/anxiety	26.9 %	19.0 %	0.470	35.0 %	23.5 %	0.363	
	Cobb angle (mild or moderate versus severe)			Modified Lenke Classification			
% in any problems	≤40° (*n* = 205)	>40° (*n* = 20)	*P*-value*	Thoracic curve (*n* = 86)	Lumbar curve (*n* = 38)	Thoracic & Lumbar curve (*n* = 103)	*P*-value*
Mobility	6.3 %	9.1 %	0.622	10.5 %	2.6 %	4.9 %	0.169
Self-care	4.4 %	0.0 %	0.316	3.5 %	5.3 %	3.9 %	0.895
Usual activities	13.7 %	18.2 %	0.562	18.6 %	10.5 %	11.7 %	0.308
Pain/discomfort	29.3 %	36.4 %	0.490	32.6 %	36.8 %	25.2 %	0.328
Depression/anxiety	19.5 %	31.8 %	0.176	23.3 %	26.3 %	16.5 %	0.337

*n* number of subjects

*Note*: * P-value of Chi-square test

## Discussion

Adolescent idiopathic scoliosis is the most common spinal abnormality in the pediatric population as seen by pediatricians and spine surgeons [43], and it can contribute to 70 % of the structural deformities affecting the spine in children and adolescents [44]. Adolescent idiopathic scoliosis patients, whether being compared to their healthy peers or comparing among different types of treatment, often have various aspects of their life being affected by the spine deformity. Therefore, it is desirable to have a reliable and suitable instrument to assess these patients’ HRQOL. The estimated HRQOL not only reflects the impact of AIS, it may also become part of the basis upon which the cost-effectiveness of differential scoliosis treatment options can be evaluated.

This psychometric validation study is the first to report the validity, reliability and sensitivity of the EQ-5D-5L questionnaire in AIS patients of Chinese ethnicity. The reliability of an instrument and whether it can reproduce consistent results is important for the assessment of the HRQOL. In this AIS population, the test-retest reliability of the EQ-5D-5L is shown to be good, despite having an ICC of less than that for SRS-22r. This is accompanied by a strong agreement for all five domains of the EQ-5D-5L (Mobility, Self-Care, Usual Activities, Pain/Discomfort, Depression/Anxiety). Being only a generic utility instrument of HRQOL, it is of utmost importance to ascertain whether the EQ-5D-5L contains the essential elements required for the assessment of the HRQOL of AIS patients. For AIS, such elements should be important for this age-group, and are tailored for how scoliosis diseases process or presentation can affect the patients.

To substantiate the validity of the EQ-5D-5L specifically for AIS, the SRS-22r was used as it is disease specific for scoliosis and contains multiple items for contributing to one domain score. Both the SRS-22r and EQ-5D-5L questionnaires are commonly-used quality of life outcome tools for patients with spinal deformity [45]. The SRS-22r is a disease-specific outcome measure commonly used for effects of treatment, and it has been used previously to assess the pre- and postoperative quality of life of scoliosis patients and the treatment outcome of bracing [46, 47], as well as being used in long-term follow-up to monitor the effects of surgery versus bracing over time or comparing untreated versus brace-treated patients [11, 48]. The EQ-5D-5L, on the other hand, is a utility measure which is more commonly used for facilitating the calculation of QALYs. This make cost-utility analysis and economic evaluations of healthcare interventions possible, such as in the study for different treatment for various diseases like those for rheumatoid arthritis [49], and juvenile idiopathic arthritis [50].

In this study, not only is the EQ-5D-5L found to have a good correlation with the overall utility score of the SRS-22r, it is worth emphasizing that the EQ-5D-5L can reflect upon certain aspects of the SRS-22r with significant strength. The EQ-5D-5L has strong correlation to the Function/Activity domain, and moderate correlations to the Pain, Appearance and Mental Health domains of the SRS-22r. With the EQ-5D-5L, those patients with “no problems” in each domain scored better in the Function/Activity, Pain and Appearance domains of the SRS-22r than those having “any problems”, hence resulting in better overall SRS-22r score. Those patients with “no problems” in the domains of Self-care, Usual Activity and Pain/Discomfort in the EQ-5D-5L are less satisfied with their scoliosis treatment than those patients with “any problems”. This demonstrates that through the SRS-22r, the EQ-5D-5L has the ability to reflect and put into context in terms of patients’ treatment with disease presentation/symptoms, despite the absence of a domain representing patients’ satisfaction with management in the EQ-5D-5L. It is worth to take into account the inherent differences of various domains being focused on by the EQ-5D-5L versus the SRS-22r, as Appearance and Satisfaction with Management in the SRS-22r cannot have comparable items in the single-itemed, generic the EQ-5D in general. At the same time, the visual analogue scale (VAS) of the EQ-5D does not have an equivalent item in the SRS-22r. The VAS is a subjective assessment of overall current health. It is of most value when looking at changes within individuals rather than cross-group comparisons [51], but may not produce health state utilities for calculating QALYs [52] with doubts in its estimation of value function [53]. Thus, EQ5D-VAS in this case may not be of great interest as the perception of general health of each patient varies, and any analysis of one score given by individuals is less conclusive as compared to the total score compounded by the five domains covered effectively by the EQ-5D-5L. Also, in studying ceiling effect, two-thirds (66 %) of patients reached the EQ-5D-5L profile of ‘11111’, suggesting a substantial ceiling effect for the EQ-5D-5L scores. Compared with previous studies in Chinese population, our results demonstrated a consistent pattern of high ceiling effect reported in the general population (78 %) [54] and other chronic conditions [55–57]. Overall, the EQ-5D-5L is found to be sensible and appropriate for the administration to AIS patients, based on the above convergent validity demonstrated from consideration of individual dimensions of the instruments.

In addition, there is further investigation into whether AIS patients with different clinical parameters or severity can be detected by the EQ-5D-5L. Both the EQ-5D-5L and SRS-22r are shown to be sensitive in differentiating clinically known groups who are receiving different treatment modalities. These include comparisons between observation treatment and bracing/surgery, and between observation treatment and bracing. Furthermore, patients with the same brace treatment can be further differentiated by the EQ-5D-5L based on the duration of bracing (less than or more than/equal to one year) with statistical significance. This is not observed with the SRS-22r questionnaire. However, the severity of spinal curvatures as denoted by the magnitude of curves in Cobb angles can only be reflected by the Mental Health domain score of the SRS-22r being worse in severe curvature (>40°) than the mild/moderate curves (≤40°). Nevertheless, the overall scores of the SRS-22r and EQ-5D-5L are not significantly different in this AIS population, according to the type and magnitude of curvatures. These results coincide with the findings by Lange et al. [48], in which there were no differences in the HRQOL-scores in patients with various types of curvature: thoracic, thoraco-lumbar, or lumbar major curve. As compared to the depiction by Mental Health domain of the SRS-22r in our results, the Self-image and Pain domain of the SRS-22 questionnaire were significantly worse in their patients with residual large curve magnitudes, but otherwise the reported the HRQOL was not related to curve size [48].

The main limitation of this study is the initial test and test-retest requiring questionnaires to be administered in different methods (test by interviews in person and retest by interviews over the phone). This is due to the routine, regular follow-up of AIS patients being more than two weeks apart, hence it is impractical to request patients to return in close succession for questionnaire interviews only. However, both the EQ-5D and SRS-22r can still have their overall scores reproduced at an acceptable and significant level. Finally, the derivation of the EQ-5D-5L score adopts an indirect two-step approach, rather than direct valuation approach using a preference weighting of the EQ-5D-5L value set. An indirect two-step approach may be subject to measurement errors in the EQ-5D-5L score, but such an approach is still the best available approach for derivation of the EQ-5D-5L scores in Hong Kong where the cultural-specific value set is not available.

## Conclusion

The EQ-5D-5L demonstrates satisfactory psychometric properties in terms of validity, reliability and sensitivity in Chinese patients with AIS. As the HRQOL of adolescents can be affected to a different extent depending on the presence of scoliosis, and differ among treatment options, it is worth utilizing the HRQOL for the assessment of the long-term outcome of various treatment modalities and their respective cost-effectiveness. In summary, our findings support the applicability of the EQ-5D-5L instrument to estimate the HRQOL and utility score for economic evaluation of treatment options for AIS patients in the future.

## Abbreviations

AIS, adolescent idiopathic scoliosis; EBM, evidence-based medicine; EQ-5D-5L, euroQoL 5-dimension 5-level; HRQOL, health-related quality of life; ICC, intra-class correlation coefficient; QALYs, quality-adjusted life years; SD,standard deviation; SF-36, 36-item short form health survey; SRS-22r, refined scoliosis research society-22; VAS, visual analogue scale.

## Additional files

Additional file 1: Hong Kong (Traditional Chinese) © 2012 EuroQol Group EQ-5D™ is a trade mark of the EuroQol Group. (DOC 82 kb) Additional file 2: SRS-22r Questionnaire. (DOC 137 kb)
