# Supplementary figures and images for: Psychometric validation of the EuroQoL 5-Dimension 5-Level (EQ-5D-5L) in Chinese patients with adolescent idiopathic scoliosis
Source: Scoliosis Spinal Disord. 2016 Aug 4;11:19. doi: 10.1186/s13013-016-0083-x (PMC4973368; doi:10.1186/s13013-016-0083-x)

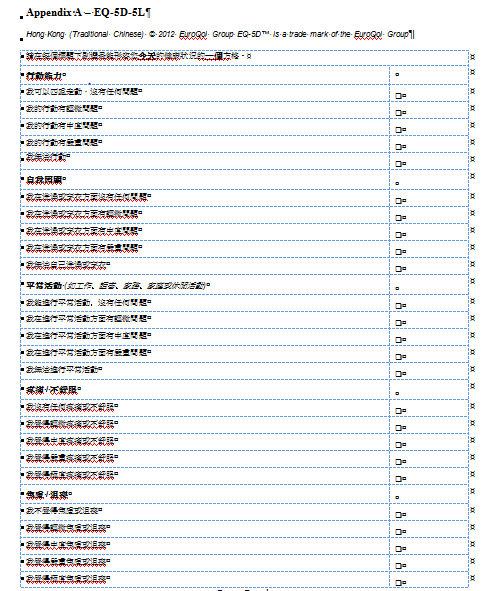


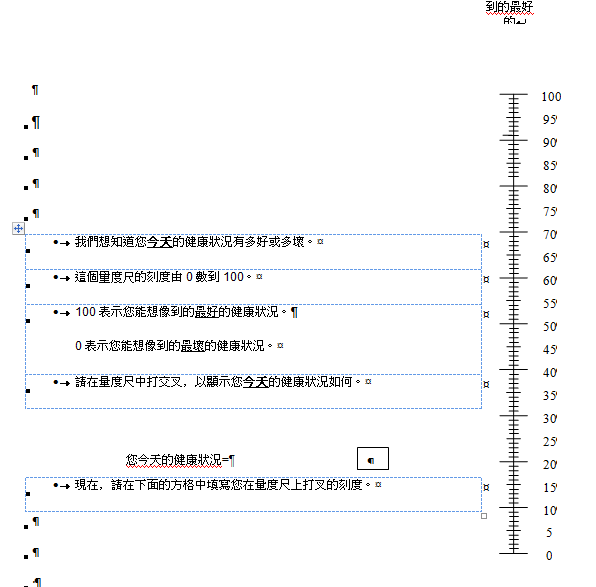

Supplement: Additional file 1: — Hong Kong (Traditional Chinese) © 2012 EuroQol Group EQ-5D™ is a trade mark of the EuroQol Group. (DOC 82 kb) [file 13013_2016_83_MOESM1_ESM.doc]

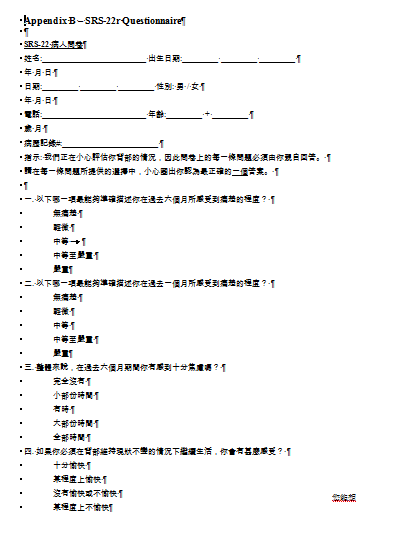


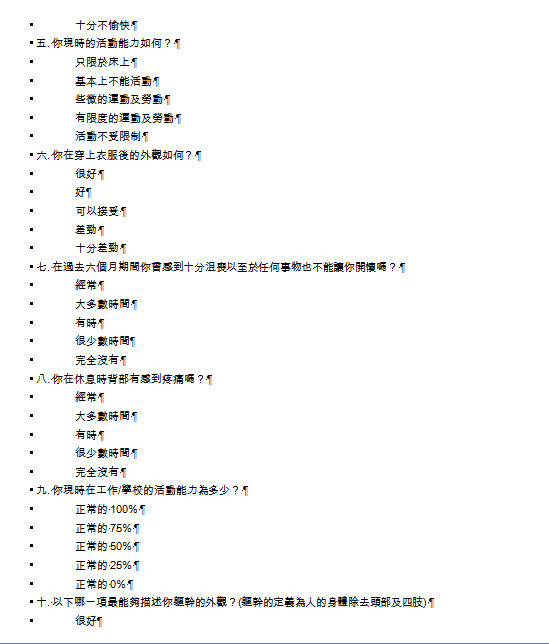


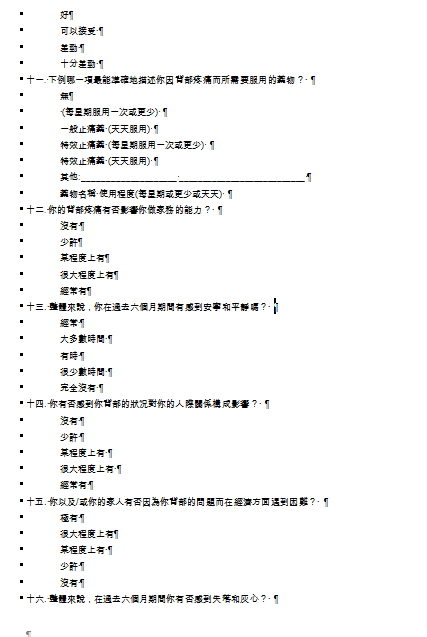


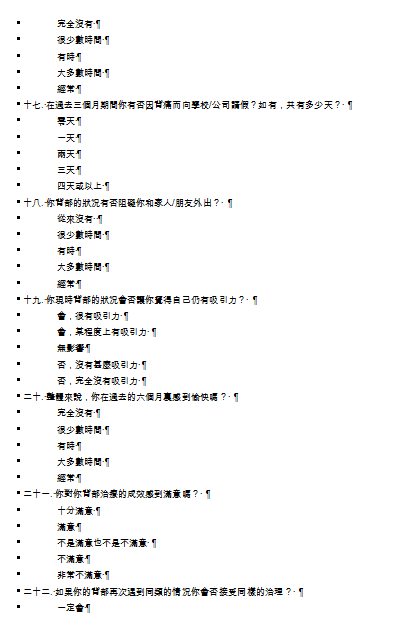


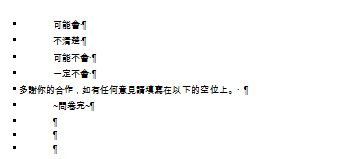

Supplement: Additional file 2: — SRS-22r Questionnaire. (DOC 137 kb) [file 13013_2016_83_MOESM2_ESM.doc]
